# Abstracts and Gleanings

**Published:** 1878-08-20

**Authors:** 


### *Abstracts and Gleanings.*

### CHLOROFORM IN LABOR.

By L. P. Yandell, M.D.,
Professor of Therapeutics and Clinical Medicine in the University of Louisville.

"The man who would whip a poor helpless woman with a cat-o'-nine-tails would receive condign punishment at the hands of the law, and, in my judgment, the doctor who allows a woman to suffer the pangs of childhood without chloroform is equally culpable, and deserves equally severe punishment." Such is the forcible language I had the pleasure to hear from the lips of the great and good Sir James Simpson, at the British Medical Association, in 1867, in Dublin. My father, professor of chemistry in the University of Louisville at the time of the discovery of the anæsthetic power of chloroform, was one of the first to manufacture this substence in the West, and was one of the earliest and most earnest advocates for its use, and, up to the time of his death, he employed it in all cases where the patient was willing to take it, and he never, in a single instance, had the slightest cause to attribute any bad result to chloroform.

Under my father's instructions, I began its administration when I entered the profession twenty-one years ago, and to-day, I entertain very much the sentiment expressed by Sir James Simpson. I have never known an untoward event to result from chloroform in labor. I know no contraindication to its use, and I advise it in every case, and give it invariably if the patient does not object.

That chloroform, in obstetrical practice, is incapable of harm, no one will assert, but its danger, like that of morphia, atropia, hydrocyanic acid, and, indeed, we may add food, water, and fire, lies in the manner of its use. Morphia, atropia, prussic acid, food, fire, and water are beneficent agents when properly employed, but, used otherwise, they may produce deplorable results. It is the excess of chloroform that does the harm when harm is done by this substance in the lying-in room.

Anæsthesia is not only necessary in labor, but, indeed, in normal cases, is only permissible in the last throes, when the acme of agony is reached, just as the presenting part is about to emerge from the vulva. By anæsthesia I mean, as the word signifies, paralysis of sensation, oblivion. Chloroform anæsthesia may sometimes produce acinesia—that is, loss of motion, or, in other words, may arrest the expulsive efforts of the uterus—but anæsthesia is not what we desire in the lying-in patient.

Analgesia—that is, absence of the sensation of pain—is the condition to be induced; only analgesia is not anæsthesia or acinesia. It has been denied that absence of pain can be compassed without the production of insensibility, and the production of insensibility, it is claimed by many, generally, if not usually, arrests the uterine contractions, or a least diminishes their strength. Such is not the fact, according to the writer's experience. In the majority of cases, even perfect anæsthesia will not arrest or enfeeble the contractions, though sometimes it does so. Analgesia, on the other hand, according to the writer's observations, may be accomplished with certainty, safety, great benefit to the patient, by the proper administration of chloroform. The recital of a case may make my meaning clearer:

Mrs. H., the mother of three children, having great fear of chloroform, had never been willing to take it in labor until she came under my charge. Her pains being intense, there was little difficulty in inducing her to inhale it, but she insisted that it should not be given after she should say, "That will do," or "I have enough." I gave it at the beginning of a severe pain, and only a few inspirations were taken when she pushed the handkerchief away, saying, "That will do." The uterine contraction expended itself in the usual time, and the patient went into a brief sleep. This procedure, with similar results, was repeated until the child was about to be expelled, when the chloroform was given freely, and total unconsciousness was produced. After a few moments, Mrs. H. awakened unaware that the child was born. She declared that, up to the last moments, she knew everything that was going on, and that she distinctly felt the expulsive efforts of the uterus, but experienced not the slightest sense of pain during their continuance. I have delivered this lady of three children, and the description of the first labor answers for all.

Mrs. H.'s case, being a typical one, sufficiently illustrates my meaning and my practice. The chloroform is best given upon a small napkin or handkerchief. Its use should only be begun after decided bearing-down pains have set in. Request the patient to give warning of the approaching pain, and when she does so, dash the chloroform, much or little, on the napkin, apply it over the mouth and nose, and direct full, strong inspirations to be made. Three or four, or half a dozen, breaths usually suffice to allay all suffering, and the expulsion movement is in nowise affected.

In France, where the comfort of women is less considered than in many other countries, the employment of chloroform in child-birth is comparatively rare, though of late it is being much more used. It has been contended by some French writer, whose name I cannot recall, that the results, such as I have enumerated in Mrs. H.'s case, are entirely due to the imagination of the patient. That such is the fact but few will believe. It is incredible that any imagination can be strong enough to render a woman oblivious to the agonies of parturition. During more than thirty years of administration of chloroform to parturient women, no death therefrom has occurred, it is asserted; but it is difficult to believe that such a statement is absolutely true, when we consider the powerful nature of this medicine and its use by so many ignorant and incompetent persons. Certainly, however, it can have done but little harm or we should have read more of its bad behavior. It is important to bear in mind, in this connection, that chloroferm is given, by most practitioners who use it, much more freely than I have recommended it. Sir James Simpson, on the occasion before alluded to, stated that, in Edinboro, he often found his patient chloroformed by the nurse when he arrived, and his practiee, and also my father's, was to keep the patient chloroformed from the beginning of the first bearing-down pains until the end of the struggle. In my own judgment, this is only necessary in abnormal labors, but in such, I have given chloroform to the degree of oblivion for twelve or more consecutive hours, and never with any unpleasant effect, unless may be a troublesome nausea or headache, which passed away within twenty-four hours.—*Louisville Med. News.*

---

### ENLARGED PROSTATE.

Dr. Atlee, in a paper read before the Philadelphia County Medical Society, on enlarged prostate, lays down the three following propositions, with remarks following:

1. That the prostate and its vessels are possessed of unstriped muscular fibre.

2. That the bladder is a hollow organ with an involuntary muscular coat.

3. That ergot will contract unstriped or involuntary muscular tissue, as it does in the uterus.

Therefore, as a corollary, ergot ought to be a remedy for enlarged prostate and its effects.

This was the theory upon which I

based the practice, and whether the rationale is correct or not, my experience in the use of ergot in such cases had been most satisfactory. Several patients over sixty years of age have been treated with ergot, and have been able to lay aside the catheter after having been the victims of its daily use. When called to a case of retention from enlarged prostate, my rule is first to relieve the bladder by means of the catheter, and follow this immediately by ordering twenty drops of the fluid extract of ergot every four hours, until the patient gets entire control over his bladder. Until this is accomplished, I continue to relieve him with the catheter every twelve hours. As his power of urination is restored, I diminish the frequency of the medicine, and gradually end in giving a dose every night. A gentleman, who died last month, at the age of ninety-two, was exceedingly ill in August, 1872, in consequence of retention of urine from enlarged prostate, and had to be regularly catheterized for relief. He was placed upon the above treatment, and in a few days was able to do without his catheter. His urinary organs were kept in a good condition by taking a dose of ergot every night, and he enjoyed much better health in consequence, and died recently of old age. I mention this case in particular, because a post-mortem examination proved to me that the prostate had been diminished in size by the treatment.

In these cases, it is very common for sedimentary deposits to accumulate in the bladder, which becomes a source of irritation and discomfort, and if the organ should fail to expel its contents entirely, it is best every few days to introduce the catheter to remove them.

---

### CASE ILLUSTRATING THE TREATMENT OF BROMIDE RASH WITH ARSENIC.

The beneficial effect of arsenic on the bromide rash deserves to be more widely known than it appears to be, and the following case, reported by Dr. Gowers (*Lancet*, June 15, 1878), illustrating it, may be of some interest. It is briefly reported from the out-patient practice at the National Hospital for the Paralyzed and Epileptic:

S. S., a man aged thirty-eight, had taken bromide of potassium certainly for five years, on account of fits, and during the whole of that time, he had had a large amount of acne upon the face. In the summer of 1877, the face was covered with coalescent acne pustules, and presented a most repulsive appearance. The eruption was also abundant on the chest. The addition of a small quantity of sulphur to each dose did a little good; the rash improved for a short time, but it soon got worse again. Sulphide of calcium was then tried, but with no further improvement, and it made him sick. The dose of bromide was then lessened from twenty to ten grains three times a day, and the acne lessened considerably, but the fits became worse, and on again increasing the bromide, the acne became more abundant, and soon was as bad as ever. On September 28, five drops of arsenical solution were given twice a day. In a fortnight, all the spots of acne were gone from the face, and those on the chest had faded. The arsenic was continued for some time, and then reduced, and ultimately discontinued. The skin remained healthy for a time, but a month afterwards the face was covered with a fresh bromide rash, red elevations, with several points of suppuration in them. Many large spots of similar kind were on the back of the neck, chest, and arms. This eruption commenced a week after the discontinuance of the arsenic. It again disappeared when the arsenic was resumed.—*Ab. Med. Science.*

---

### UNOFFICINAL PLANTS.

American Ivy. The bark and twigs have been used for dropsy.

American Senna. Analogous to senna, and contains a principle similar to cathartin. Efficient and safe cathartic, though less active than the imported senna.

American Water Hemlock. Closely analogous to European species. In several instances, children have been fatally poisoned by eating its roots. It is highly recommended as a specific in nervous headache. The seeds contain an alkaloid supposed to be identical with conia.

Life Everlasting (Antennaria Margaritacca). The leaves are astringent and expectorant.

Cashew-nut Tree (Anacardium Occidentale). A small tree of the West Indies, from the bark of which a gum exudes, similar to gum Arabic, but only in part soluble in water. Its fruit affords a juice which has been recommended in uterine complaints and dropsy.

Aristolochia. Fourteen species have the same medicinal virtues as the officinal A. serpentaria.

Pleurisy Root possesses diaphoretic and expectorant properties, without being a stimulant.

Ascletine, an "eclectic" preparation, recommended as the active principle of pleurisy root.

Beaked Hazel, the nut of which is covered with short spiculæ, which have been used, like cowhage, as a vermifuge.

Beech Oil is used in Picardy, and other parts of France, instead of butter.

Bladder-wrack (Fucus Vesiculosus). A sea-weed growing on the shores of Europe. It contains soda, in saline combination, and iodine, in the state of iod. of potash. Used to decrease obesity.

Bouncing Bet is a vulgar name for soapwort.

Broom-rape has been used in cancerous affections.

Bugle-weed. Mild narcotic and astringent.

Camellia Sasanqua. An aromatic plant, the flowers of which are sometimes mixed with tea leaves in order to render them fragrant.

Cardinal Flower (Lobelia Cardinalis). A species of lobelia, said to possess anthelmintic properties.

Cassina. The leaves possess emetic properties, and form, in decoction, the black drink of the Indians.

Castanea. The bark of castanea pumila, a species of chestnut tree, the bark of which is astringent and tonic.

Ceanothus Americanus (New Jersey Tea, Red Root). Root is astringent, and said to be useful in syphilitic complaints. The leaves have been used as a substitute for tea. Ceanothine is a name given to an extract prepared from the leaves of this plant.

Celandine (Chelidonium Majus) possesses diuretic, diaphoretic, and expectorant properties, and said also to be an acrid purgative.

Celastrus Scandens (Climbing Staff Tree), the bark of which is said to possess emetic, diaphoretic, and narco properties.

Yellow Dock. Astringent, tonic, and alterative.

Common Elder, the flowers of which are gently excitant and sudorific.

Ginseng, a favorite remedy with horse doctors, is little more than a demulcent.

Horse Mint is stimulant and carminative.

Mountain Ash. All parts astringent.

Water Pepper. Diuretic.

Barberry. A cooling drink is made of it in New England for fevers.

True Watercress. Anti-scorbutic.

Prickly Ash. Stimulant emmenagogue.

Buckthorn Family. Violent cathartics.

Common Geranium. Root is astringent; may be boiled in milk and sugar; excellent for children. The root should be collected in autumn.—*Rothrock.*

Common Wild Indigo. Used in domestic practice; also, in eclectic practice. Some authorities say it is very useful in typhoid fever, used in place of turpentine—that is, for just such symptoms as require the use of turpentine.

Quince. Seed astringent and demulcent.

Blessed Thistle. Harmless; simply mucilaginous.

To those camping out, the Common Hemlock makes a good tea.

The Common Hop is widely distributed.

Wild Hop can be used with perfect safety.

Butternut is much used by the eclectics; it is a good laxative; also, good for habitual constipation, not creating secondary costiveness, like rhubarb.—*Philadelphia Drug. and Chem.*

---

MEDICO-LEGAL EVIDENCE.

Prof. Joseph Jones having pronounced the stains found upon the clothing of a suspected murderer to be caused by human blood from a person suffering from malarial fever, was thus questioned by the defence before the court!

EXAMINATION ON THE PART OF THE DEFENCE.

D. Are you absolutely certain that the stains on the pieces of cloth, placed in your hands for microscopical and chemical analysis were caused by human blood?

J. The substances causing the stains presented all the chemical and microscopical properties of human blood, or blood presenting a special pathological alteration.

D. Can you by means of chemical and microscopical examination of the blood determine any form of disease?

J. By chemical and microscopical examination, I am not able to determine every form of disease.

D. You would then be in doubt concerning the result, in certain diseases, of the chemical and microscopical examination; please state therefore if there are any diseases, the nature of which may be revealed by the microscope?

S. The microscope enables us to distinguish clearly the change induced in the blood by malarial fever. That condition of the blood known as leucocythaemia or leukaema can be accurately determined by microscopical examination. There was no doubt in my mind that the blood examined was human blood from one who had suffered and perhaps was at the time suffering with malarial fever.

D. What did you say was the average size of the colored blood corpuscles in the stains upon the cloth? and whilst giving this measurement, give those also of the dog, horse, rat, cat, rabbit, ass, ox, cow, pig, sheep and goat.

S. The average of the diameter of the blood corpuscles from the stains, was about 1-3200 of an inch. The corpuscles of human blood are larger than those of domestic animals. Thus the average diameter in the dog, is about 1-3540 of an inch; horse, 1-4600 of an inch; in the rat, 1-3814 of an inch; in the cat, 1-4400 of an inch; in the ox, 1-4267 of an inch; in the ass, 1-4000 of an inch; in the cow, 1-4200 of an inch; in the pig, 1-4230 of an inch; in the sheep, 1-5300 of an inch; in the goat, 1-6366 of an inch.

D. Do not those individual blood corpuscles in man and animals vary in their diameter in the same specimen of blood? and if so, state to the honorable court the causes of these variations.

S. The blood corpuscles of man and animals vary in their diameters within their certain limits; thus those of man may vary from 1-2000 to 1-4000; of the dog from 1-4000 to 1-6000; of the hare from 1-2000 to 1-8000; in the ox from 1-4878 to 1-4444; in the sheep 1-5333 to 1-6000 of an inch.

D. Difficulty therefore exists in distinguishing between the blood of man and domestic animals; and in view of this fact do you assert *absolutely*, that the stains in the pieces of cloth were human blood?

S. I admit that difficulties exist in such examinations. I affirmed that the human blood corpuscles upon an average were larger than that of the domestic animals named. I also affirmed that the stains upon the pieces of cloth presented all the characteristics of human blood.

I went a step further and affirmed that this blood presented pathological appearances which as far as my investigations extend, are peculiar to human blood in a certain diseased state, and that I have never observed such a condition in the blood of animals; and that the blood from the house in which the deceased was murdered presented similar chemical and microscopical characters.

D. Can the colored blood corpuscles be detected with accuracy in dried blood?

S. They can be detected in many cases; and they were detected accurately in the case now before this honorable court.

D. Can you distinguish between the blood of a woman and the blood of a man?

S. I cannot.

D. Can you distinguish the blood of a fœtus from the blood of its mother?

S. I cannot.

D. Can you distinguish the blood of the different races of men? for example, can you chemically and microscopically distinguish the blood of a white man from that of a negro?

S. Different races are said to have distinct odors; sulphuric acid applied to blood will liberate the peculiar odor of the animal; I have upon many cases satisfied myself of the possibility of developing the peculiar odor of the blood in different animals by means of sulphuric acid. I cannot, however, speak positively with reference to the blood of the different races of mankind.

VERDICT OF JURY.

By the testimony of several witnesses, two of whom were practicing physicians, it was clearly established that, for some weeks before and up to the time of his murder, Narcisse Arrieux was suffering with intermittent malarial fever (chills and fever). The judgment of the jury rested to a great degree upon the presence of blood on the clothing of the accused Wilson Childers.

We have been informed by Mr. John H. Ilsley, jr., one of the attorneys for the prosecution, that an important witness testified as to the guilt of the four negroes accused of the murder of Narcisse Arrieux. The jury rendered the verdict guilty of murder with capital punishment.—*O. N. Med. Jour.*

SUBSTITUTE FOR MOTHER'S MILK.

Dr. Marsh, in the *Obstetric Gazette*, says:

If we mix three parts of cow's milk with one part of water, and add about an even teaspoonful of sugar of milk to a half pint of the mixture, we will have about the proportions indicated in the table for human milk. Cane sugar may be substituted for sugar of milk, but is not so good probably. Most authors recommend the addition of a still larger proportion of water for the first few weeks of infantile life, gradually decreasing the quantity of water as the child grows older, until it is able to take the milk undiluted. If the curd is not readily digested, the addition of an alkali will often render it more digestible. For this purpose, carbonate of potash in the proportion of one grain to the fluid ounce may be used, or, which is preferable, use lime water instead of water alone to dilute the milk. Lime water contains about half a grain of lime to each fluid ounce, and should be prepared and kept as follows: The lime should be freshly burned, and, to insure this, it is better to put a piece in the fire and let it remain an hour or two. About an ounce of this should be slacked by pouring on a sufficient quantity of water, and, when thoroughly slacked, put it in a quart bottle, fill the bottle with cold rain water, shake it well and let it stand until the lime settles, when it is ready for use. The bottle should be kept well corked, and the lime allowed to remain constantly saturated. Care should be taken in pouring the lime water off for use, that none of the lime in the bottom is allowed to escape and be mixed with the food. The milk should be fed from a suitable feeding bottle, so constructed that the child cannot suck and swallow air with its food, as this causes great uneasiness. "Maw's Feeding Bottle" is the best. The milk should not be boiled, but the bottle containing it should be placed in hot water until the feed acquires a temperature of 98° F. If any milk remains in the bottle at the end of the meal, it should be thrown away. The bottle should be thoroughly cleansed after each meal. Not the least particle of milk should be allowed to remain adhering to any part of the apparatus, and the caps and tube should be kept in cold water slightly alkalinized. This precaution is especially necessary in warm weather. The milk should be obtained fresh every morning and evening, and kept at a temperature of about 40° F., and each meal freshly prepared. It should be obtained from a young cow whose calf, if living, should be from two to six months old. The child should be taught to take its food at regular intervals. For a child six weeks old or under, three or four ounces every two or three hours; as the child grows older, the quantity may be gradually increased and the interval lengthened. The child should also be taught to take its last meal for the day at about 10 o'clock P.M., and the first at about 5 o'clock A.M. This will insure, for both child and nurse, a good night's rest, which cannot be secured if the child is in the habit of waking frequently in the night to be fed. We do not think it advisable to add anything to this simple milk diet, if the child continues to do well on it, until after the seventh or eighth month, when some farinaceous element may be added, such as bread or crackers, broken up and thoroughly soaked in the milk, or rice, sago, tapioca, arrow-root, farina, etc., thoroughly and carefully cooked, and only in such quantities as can be entirely digested. Sometimes "Liebig's Food for Infants" agrees better than any of these. This consists of milk, wheat flour, and malt, with a little bicarbonate of potash. The object of the malt is to convert the starch into grape sugar, and thus relieve the digestive organs of part of their labor. In London, this food is freshly prepared in the liquid form every day, and is sold for about six pence a quart. It is also prepared in a concentrated form, and a dry preparation by Messrs. Savory & Moore may be obtained in this country. As this paper does not comprehend the diet of infants after the proper age for weaning, it will not be in place to speak of such articles of food as may be given after that age.

---

### TREATMENT OF ACNE ROSACEA.

Professor Hebra, of Vienna, reports six cases of acne rosacea, with the view of illustrating the different modes of operative treatment that may prove serviceable against this affection. Two of the cases presented the first degree of the affection, namely, cutaneous redness with dilatation of the vessels. These were treated by repeated punctures, which brought about the obliteration of the dilated vessels. The instrument used was a needle with a lance-shaped, two-edged blade, about one-twelfth of an inch long, and provided with a shoulder to keep it from penetrating too deeply. In performing the operation, Prof. Hebra stands behind the patient, who is seated in a chair, with the head bent backwards. He makes the punctures as close together as possible, and with the rapidity and regularity of a sewing machine. At each puncture, the needle is driven in until it is arrested by the projecting shoulder with which it is provided. If possible, the entire morbid tissue should be operated upon at a sitting. The hemorrhage is always considerable, but it is readily controlled by compression. The dressing consists of dry charpie secured by a bandage, and it should be renewed daily. Should suppuration occur, which is rare, the dry dressing must be replaced by lead ointment or simple cerate. Sometimes it is necessary to repeat the operation several times before a cure is effected.

Two of the six cases presented, in addition to the telangiectasis, considerable thickening of the cutis and numerous pustules. They were treated by scraping with the sharp curette. Although the curette only scrapes off the epidermis, the operation apparently causes obliteration of the vessels, as it is followed by the disappearance of the redness and thickening. Not unfrequently, it is true, this result is only obtained after repeated operations and the development of superficial suppuration. Superficial nævi have sometimes been destroyed by a similar application of the curette. In the two cases reported, the operation was entirely successful.

Finally, the two remaining cases presented the third degree of acne rosacea, namely, great thickening of the skin of the nose, with the development of fleshy excrescences. These were treated by the knife, the protuberances and hypertrophied parts being cut off or trimmed down. In one of the cases, a fleshy mass, which extended nearly an inch beyond the nasal cartilage, was removed by the elastic lagature for fear of hemorrhage, but Prof. Hebra states that in a similar case, he would not hesitate to remove the mass at once with the knife. In each case, he succeeded in forming a very presentable nose, to the great delight of the patients and his own satisfaction.—*Wiener Med. Wochenschr.*, Jan. 5, 1878.

HYSTERIA IN A BOY.

Dr. Warren, in the North Carolina *Medical Journal*, says:

I was recently called to a case of hysteria in a boy of twelve years, which had existed for sometime without having been recognized by his regular medical attendant. Finding the symptoms urgent, I gave him injections of chloral in solution, and applied ice to the spine, with the result of promptly restraining the paroxysms. During my absence, at a subsequent period, he became again convulsed, when, instead of resorting to the remedies which had been previously employed, his attendant made the attempt to quiet him by the forcible administration of chloroform. Instead of the desired anæsthesia, a fearful delirium—an excitation of the most aggravated and distressing character—was induced, which continued for several days and precluded a recourse to the treatment which had originally proved so successful. In this emergency, I addressed a note to Professor Charcot, requesting a consultation at the earliest possible moment. As an act of special courtesy to an old friend, he came on the succeeding day at a late hour, although to do so he had to disregard engagements which would have required, for their fulfillment, an entire week—such is the demand for him at the present moment in Paris. After making a thorough examination of the patient, he agreed to the correctness of the diagnosis already made, and recommended a course of hydrotherapy in an establishment especially devoted to that mode of treatment. As you are doubtless aware, Charcot believes that the sovereign remedy for hysteria, in all of its phases, is cold water, together with the removal of the patient from the scenes and persons in connection with which, and with whom the disease has been primarily developed. His advice was followed in this instance, and the little fellow began to improve at once, and is now comparatively well. I have referred to this case, not so much because of any special attention which attaches to it, but rather for the purpose of introducing the great man whose experience and skill I was so fortunately able to invoke in behalf of my patient. As a neuro-pathologist, in the broadest and highest sense of that term, Charcot stands *primus inter pares* in the medical world of France. He is, in fact, the great authority to whose dicta the profession bows in unquestioning reverence, and in whose career it feels the deepest interest.

Dr. Valquardsen reports, in Schmidt's Dictionary and the *Pesth Medico-Chirurg. Presse*, No. 39, 1877, a case of sciatica, which lasted for two years, and defied all treatment. He then arrived at the idea of trying the internal use of phosphorus, which he prescribed in doses of fifteen milligrammes (about one-fourth of a grain) three times a day. Three days sufficed to obtain a marked improvement, and three weeks brought a complete cure.—*Lond. Med. Record.*

### THEORY OF THE ACTION OF QUININE.

Dr. F. W. Moinet, at the close of a careful article on this subject, in the Edinburgh *Medical Journal*, says: Arsenic, caffeine, alcohol, beberia, piperine, gentian, capsicum, strychnia, etc., although differing considerably both in kind and degree of active power, have some power over intermittent fever and other diseases of a nervous pathology. In fact, as adjuvants, and occasionally as substitutes, they are invaluable, as clinical experience has amply testified. In virtue, however, of certain peculiar properties, one is more useful than another in different nervous diseases, due partly to their somewhat different actions, the part of the nervous system affected, and the cause of the disturbance. Thus, quinine appears to exert an influence on that part of the nervous system for which the malarial poison has a special affinity, and, in virtue of this, is more curative than the other remedies, just as arsenic has a special tonic influence on the motor nerves, in virtue of which it is more powerful in chorea, and caffeine, an action on the pulmonary nerves, which renders it more useful in asthma; hence, we believe quinine to act in malarial diseases as a stimulant or sedative to the nervous system, especially to that part most implicated in these diseases, and that it is principally in virtue of this action that it proves curative by rendering the malarial poison inoperative by an antagonistic action on the nervous system, and that it proves beneficial in proportion as the nervous disturbance is predominant, and to the absence of complications. Although we arrive at this conclusion by argument, and not direct experiment, the evidence in its favor appears to us so strong as to give it the place of a more than probable theory, and to be a much more reasonable explanation of its action than any other as yet brought forward, and that, by this method of reasoning, it is possible to arrive at correct conclusions, in absence of direct experience, the literature of therapeutics amply shows.—*Med. & Surg. Rep.*

### LUPUS.

Dr. Taggart, of Arizona, reports the following case of Lupus in *Pacific Medical Journal*:

John Brown, aged forty-five, and in good general health, called on us December 1, 1877, for treatment for an ulcer on the left side of the nose, extending from near the middle line to within one-fourth of an inch of the inner canthus. It was this near approach to the eye that induced him to seek assistance. The ulcer first appeared about fifteen years since as a pimple near the point of the nose, and refusing to heal, slowly advanced upward, the skin over which it passed resuming its natural appearance. Sometimes the ulcer would seem to be almost entirely well, only a dry scab apparently being left, but always recovering itself and resuming its march. Some two years since it arrived near its last position, where it was attacked with caustic, presumably nitrate of silver, by a physician under whose care he then was, but it refused to heal, and it was noticed, after the inflammation incident to the use of the caustic had subsided, that it was deeper than before, and would sometimes be visited by burning or stinging pains. We found the ulcer elliptical in shape, the greater diameter horizontal and about three times as long as the other, its cavity filled with pale, sickly-looking granulations. The first application was of zinci chlor.; sanguinariæ pulv.; glycyrrhizæ pulv., equal parts, mixed with water to a paste. The ulcer was filled with this preparation, which was not removed till next day. Very little pain followed. When the caustic was removed, a poultice of slippery elm was applied. In about three days the eschar separated in a mass, leaving a healthy sore. In less than two weeks it was well. The only mark left is a firm, linear cicatrix about an inch in length.

---

### INTERTRIGO IN CHILDREN.

Dr. Wertheimer recommends the use of corrosive sublimate (gr. i to oz. iijss of water) for the intertrigo of children, as the most effective remedy.

### CHLORAL IN DYSENTERY.

Dr. Wm. L. Newell, in the *Phil. Med. Times:* A weak solution of that valuable medicine on chronic ulcers manifested such favorable results in my hands that I conceived the idea of using it locally on the inflamed and congested bowel in dysentery. Ti e first case had been under the usual treatment for three days without relief. The child, aged eleven, was tormented with thirst, pain and tenesmus, with twenty-five or thirty dejections in twenty-four hours. In connection with other treatment I ordered five grains of chlor. hyd., dissoved in two ounces starch gruel, thrown up the bowel with considerable force from a hard rubber syringe. It remained three hours, during which the child slept. Many of the other symptoms were modified, and the injection was repeated, which remained seven hours, when it came away with some fecal m tter. but without tenesmus. The child asked for food, which was given in the form of mutton tea thickened with boiled wheat flour. All treatment ceased in forty-eight hours from the first enema, four being given in all. The case seemed so satisfactory that I mentioned it to my confrere, Dr. J. S. Whitaker, who has pursued the same treatment with the most happy results in every case, aborting the disease within a few hours. I may mention that he used ten grains instead of five with a lady, aged twenty-five years, who had twenty or thirty calls in twenty-four hours, with complete repose for eight consecutive hours, with permanent abatement of all other symptoms, without other treatment. — *Luisville Med. News.*

---

### A NEW TREATMENT OF TAPE-WORM.

From the results of numerous experiments, M. Bouchut had ascertained that not only ascarides, but fragments of tænia, when placed in a weak alcoholic solution containing one thirty-fifth of amylaceous pepsine, are digested by the fluid in the course of twelve hours. We thus obtain an artificial digestion of the animal matter exactly similar to that which ensues when meat is treated by the same process. On submitting the conclusion drawn from his experiments to the test of practice at the Enfans Malades, M. Bouchut found that the solution of pepsine was eminently successful. If his experience be confirmed, a valuable addition will be made to adult as well as infantile therapeutics. In conclusion, we may observe that animal food is, almost certainly, the channel through which the parasite is conveyed; and hence that officicl inspection of suspected dealers in meat would form a useful adjunct to the practice of the physician.—*Ib.*

---

### MORPHLOGICAL CHANGES IN SYHILITIC BLOOD.

In the Section on Practical Medicine, at the American Medical Association:

"Dr. Ephraim Cutter, of Boston, gave a micro-photographic exhibition of the morphological changes which occurred in the blood in consequence of syphilis. The diagnosis of syphilis was based upon the presence in the blood of a copper-colored filiform growth with rounded and enlarged extremities and spores. Dr. Cutter regarded the demonstration as corroborative of the claims made by Dr. Salisbury in connection with the same subject.

"He thoroughly believed that positive diagnosis of syphilis could be made by microscopical examination of the blood.

"He also believed that Lostorfer was correct in his statement that the white blood corpuscles were enlarged in syphilis; but he should have insisted upon the copper color of the spores."—*Ex,*

---

### PERSISTENT HICCOUGH TREATED SUCCESSFULLY BY PILOCARPINE.

Dr. Ortille, of Lille, writes to the *Bull. Gen. de Therap.*, 1878, p. 412, giving an account of a case of obstinate hiccough, in which, after trying all the usual remedies, he had recourse to electricity, which was found so successful by

Dr. Dumontpallier. For a few hours, the electrical applications appeared to prove successful, but the hiccough returned. Dr. O. was about to apply electro-puncture, with a view of tetanizing the diaphragm, when, recollecting what he had read of the action of pilocarpine upon the phrenic nerves, and of the vomiting which so often follows its use, he injected two and one-half centigrammes (two-fifths of a grain) of pilocarpine under the skin. The effect was surprising and almost instantaneous. A quarter of an hour after the injection, the patient was covered with sweat, salivation was established, and the hiccough had ceased, not again to recur.—*Ib.*

### THE USE OF OLEATE OF MERCURY IN EYE DISEASES.

M. Landesberg, M.D., in the *Medical Times*, says:

From an experience extending over several months, gained in a large number of conjunctival and corneal affections, which I treated according to the same indications with the new preparation as formerly with the yellow oxide of mercury, I am fully justified in saying that the oleate of mercury has all the qualities that will render it fit to supplant entirely the former preparation in oculistic practice.

The oleate of mercury is mixed very easily with cosmoline, undergoes no decomposition or rancidity, and remains for any length of time without the slightest alteration. It may be prepared in its proper form by any skillful pharmacist. The ointment presents a yellowish, diaphanous appearance of slightly firm consistency. Brought between the eyelids, it readily melts, and can be rubbed in so completely that not the smallest particle remains. Its capability of assimilation and absorption is very great. The reaction of the eye upon the application is but inconsiderable, less than upon the use of the yellow oxide ointment.

The only precaution to be observed by the pharmacist, in preparing the oleate, is to see that the oleic acid be pure, and that it be recently prepared with fresh oil of sweet almonds.

### A CASE OF HEMORRHAGIC COXITIS CURED BY PUNCTURE.

C. Langenbuch, in the *Deutsche Zeitschr. f. pract. Med.* (*Cbl. f. Med.*, 1878, p. 285), gives the case of a seventeen-year-old otherwise healthy girl, who, after an attack of polyarthritis, suffered great pain and swelling in the left hip-joint. A puncture undertaken with antiseptic precautions gave exit to some one hundred centimetres (three ounces) of a bloody fluid. The whole limb was put up in a plaster-of-Paris bandage, pain ceased at once, and a cure was effected within a few weeks.—*X. in Med. Times.*

### NITRATE OF SILVER INJECTION IN CHRONIC DYSENTERY.

Dr. H. C. Wood strongly recommends the solutions of nitrate of silver in the treatment of chronic dysentery. He dissolves from forty to sixty grains of the salt in a quart of warm water, which he then throws into the bowels by means, preferably, of a gravity syringe, which enables the fluid to reach a high point in the affected colon. The injection is usually retained from five to fifteen minutes, and may be repeated daily, or less often, as the case may demand.—*Phila. Med. Times.*

### TREATMENT OF CANCER BY PRESSURE.

This is the very latest novelty in the treatment of this disease. M. Bouchui has invented a cuirasse of vulcanized india rubber, which he is said to have used with success for the treatment o cancerous and other tumors of the breast. The idea is not new, experiments having been tried in this direction without success. An analogous method is the ligature of the nutrient artery of such a growth, as in disease of the tongue.

The 'Lancet' remarks that it is obvious if pressure is to be effective it must be applied around the periphery of the growth, where the cell proliferation is most active. This must be obtained, it is said, by the careful adjustment of pads of cotton-wool. The neatest plan would seem to be the employment of compressed sponges, which might be bandaged firmly around a tumor of the breast, and then allowed to swell by imbibition of water. The constriction of the chest would. of course, be great and thoracic respiration seriously interfered with. But the patient might be kept in bed, where abdominal respiration might suffice.—*Boston Med. & Surg. Jour.*

---

TREATMENT OF REMITTENT EEVER.

Dr. Clay, in the "Nashville Medical Journal," reports a case of bilious remittent fever as follows:

R.—1. Hyd. chlo. mit.................... grs. 10.
To be taken in broken doses and followed with castor oil.

R.—2. Pulv. opium........................ grs. j.
Gum camphor........................ grs. i.
To be taken every four hours.

R.—3. Tr. veratrum viridi............ gtt. ijss.
Aconite root........................ gtt. ijss.
The quantity to be taken every two hours during exacerbations.

R.—4. Sulphate quinine.................. grs. 10,
every four hours during remissions.

Cold applications to head, sponging, cold drinks freely, good ventilation.

In twenty-four hours the bowels had moved gently. Patient expressed himself as feeling much better, with some appetite. Temperature, almost normal; pulse about the same as during remission. Urine highly colored, but no hemorrhage.

*Treatment.*—Discontinued use of sedatives and mercury, lessened quantity of opium and camphor. Patient gradually improved, and on the fourth day of my attendance was dismissed, cured.

---

CHLORAL AND GLYCERINE IN CONSTIPATION.

A patient of a medical friend discovers that chloral solution relieves habitual constipation. He had been injecting chloral solution, 10 grains to 1 ounce, with a bulb-pipette into the rectum, and externally to the anus to relieve pruritus of the parts, when he discovered that he had a prompt evacuation of the bowels. Thinking it an accident, he repeated it often enough to convince himself and his physician. Dr. Hanson, of Davison's Station, Michigan, uses one part of glycerine to six, eight or ten parts of warm water to produce the same effect. *N. C. Med. Jour.*

---

THIERSCH ON CAUTERIZATION OF NÆVI.

London *Medical Record:* Dr. Thiersch applies over the surface of the tumor a little plate of copper, pierced at regular and small distances with small holes. Through these he passes a needle mounted in a cork, and previously heated in a spirit lamp. The cauterization is thus effected very regularly. The same method is applicable to the liner division of the skin by a cutting needle, recently recommended by Mr. Balmanno Squire.—*Ib.*

---

JABORANDI IN OBSTINATE HICCOUGH.

Dr. Ortille, of Lille, relates (*Bull. de Therapeutique*, May 15) a case of most obstinate hiccough, in which he had tried a great variety of means, including electricity and hypodermic morphia injections—the hiccough even continuing during the sleep caused by this last. He then tried the hydrochlorate of pilocarpin on account of its acton on the phrenic nerve. A hypodermic injection of two centigrammes and a half was inserted with almost immediate effect, so that in a quarter of an hour, the patient was bathed in sweat, salivation was established, and the hiccough disappeared, never to return.—*Med. Times and Gaz.*

---

Dr. Chenoweth, in the Louisville *Medical News*, says: "I do not not hesitate to employ chloroform in the first stage of labor, which the presence of a rigid os promises to render tedious. I give it in the last stage of labor, which uterine contraction and a resisting perineum render more than ordinarily painful.

